# Notes and News

**Published:** 1904-06-18

**Authors:** 


### NOTES AND NEWS.

THE King has consented to lay the foundation stone of the new buildings at St. Bartholomew's Hospital on July 6th.

THE Queen has consented to become President of the London Hospital.

THE Princess Christian has become president of a ladies' committee which will appeal for assistance to build a sanatorium for consumptives at Davos, of which the Queen is Patron, and which will be called The Queen Alexandra Sanatorium. In this sanatorium each patient will have a separate room, and accommodation will be provided for about 40 to begin with. The site of five acres is 5,400 feet above sea-level. The initial cost is estimated at about £12,000, and it is hoped that when once the value of the institution is realised, that considerable enlargement will follow.

LORD WOLVERTON will preside at the festival dinner of the Metropolitan Hospital at the Hôtel Métropole on June 23.

AT the International Home Relief Congress at Edinburgh a discussion was opened on the relation between hospital and home relief of the sick poor. Sir Henry Burdett, K.C.B., being unable, owing to his severe accident, to open the discussion, Dr. Bruce of Dingwade acted in his place; but as he advocated rate support for hospitals, the line of discussion was on different lines from what it would have been, though his views on the abuse of the out-patient department and other points were in accord with our own.

A GOOD deal of discussion and divergence of opinion has taken place at the Cumberland Infirmary on the question of utilising a verandah for the outdoor treatment of cases likely to benefit. The medical staff are in favour of this course, but some of the lay management have opposed it mainly for administrative reasons. It seems to us the question can be very simply answered. If cases need a certain method of treatment recommended by the medical staff, the Cumberland Infirmary should either carry out the recommendations, or not receive such patients.

IT is reported that the plague is spreading rapidly at Payta, in Peru, and that the percentage of mortality is heavy.

THE late Mr. Frederick Gordon, of Stanmore, has bequeathed £1,000 to King Edward's Hospital Fund and £500 to the Surgical Aid Society.

THE new Sanatorium for the Brompton Hospital for Consumption will be opened at Camberley on June 25th by the Prince and Princess of Wales.

THE Festival Dinner of the Royal Waterloo Hospital for Children and Women will be held at the Savoy Hotel on June 20th. The dinner is by special invitation only, and will be served at separate tables.

THE prize distribution at the medical school of St. Thomas's Hospital will be held in the Governors' Hall of the hospital on Friday, June 24, at 3 P.M. Sir Thomas Barlow, Bart., K.C.V.O., M.D., will give an address, and a garden party will follow in the grounds.

THE largest amounts received by the Metropolitan Hospital Sunday Fund from the collections in the churches include:—St. Paul's Cathedral, £4,520; St. Michael's, Chester Square, £1,376; Theistic Swallow Church, Street, per the Rev. Charles Voysey, £313; and the total to date exceeds £10,000.

THE Bill authorising St. Bartholomew's Hospital to demolish St. Bartholomew's the Less, and to utilise the site and burial ground attached for purposes of the hospital has been ordered to be reported to the House of Commons without amendment by the Committee on unopposed Bills.

THE family of the late Mr. David Cargill, of Glasgow, knowing his intention to benefit the charities, are devoting £20,000 to fulfil his wishes. Through this munificent gift the Glasgow Royal Infirmary will receive £2,000; the Glasgow Western Infirmary, £2,000; the Glasgow Victoria Infirmary, Cancer Hospital, and Maternity Hospital each £1,000; and £500 each will be given to the Arbroath Infirmary; Royal Hospital for Sick Children, Garnethill; the East Park Cottage Home for Infirm Children; the Association for the Relief of Incurables, Broomhill; and smaller sums to numerous medical and other charities.

A COUNTY Hospital Work Guild has been formed for the benefit of the Lincoln County Hospital. The scheme has been modelled upon the lines of similar organisations existing in connection with other country hospitals, and is intended to replenish the entire linen and bed furniture in the hospital, as and when required. It is formed amongst the ladies of the neighbourhood. The idea seems to us an excellent one, and worthy to be universally adopted. People are usually much more inclined to give of their time and money for some definite object, and doing it in this manner a general interest in the well-being of the institutions is stimulated. Personal service is far more valuable for our hospitals than even a generous contribution paid often automatically by the donor's banker.

